# Granular cell ameloblastoma of jaw – Report of a case with an emphasis on its characterization

**DOI:** 10.4317/jced.51015

**Published:** 2013-07-01

**Authors:** Sravya Taneeru, Venkateswara R. Guttikonda, Sivaranjani Yeluri, Jayakiran Madala

**Affiliations:** 1 M.D.S., Post Graduate Trainee. Department of Oral pathology and Microbiology, Mamata Dental College and Hospital, Giriprasadnagar, Khammam, Andhra Pradesh, India; 2 M.D.S., Professor and Head. Department of Oral pathology and Microbiology, Mamata Dental College and Hospital, Giriprasadnagar, Khammam, Andhra Pradesh, India; 3 M.D.S., Reader. Department of Oral pathology and Microbiology, Mamata Dental College and Hospital, Giriprasadnagar, Khammam, Andhra Pradesh, India; 4 M.D.S., Senior Lecturer. Department of Oral pathology and Microbiology, Mamata Dental College and Hospital, Giriprasadnagar, Khammam, Andhra Pradesh, India

## Abstract

Ameloblastoma is a neoplasm of odontogenic epithelium, especially of enamel organ-type tissue that has not undergone differentiation to the point of hard tissue formation. It accounts for approximately 10% of all tumors originating from gnathic bones. It exhibits diverse microscopic patterns which occurs either singly or in combination with other patterns. Granular cell ameloblastoma is a rare condition, accounting for 3.5% of all ameloblastoma cases that shows marked transformation in the cytoplasm of tumor cells, which are usually stellate reticulum like cells. The transformed cells possess very coarse, granular, eosinophilic cytoplasm. The “granular change” is thought to be due to a dysfunctional status of neoplastic cells, and the pathogenesis of this tumour seems to be age-related. Ultrastructural, histochemical, and immunohitochemical studies have revealed that cytoplasmic granularity is caused by overload; however the mechanism ivolved remains poorly understood. This article describes a case of granular cell variant of ameloblastoma affecting a 55-year old female.

** Key words:**Ameloblastoma, granular cell, odontogenic tumor.

## Introduction

Odontogenic tumors (OT) are a group of heterogenous lesions derived from epithelial and/or mesenchymal elements that are part of the tooth-forming apparatus (1). Ameloblastoma is well recognized as a locally invasive benign neoplasm thought to arise from the cellular components of the enamel organ (2). It is an epithelial odontogenic tumor of jaw and exhibits diverse microscopic patterns which occurs either singly or in combination with other patterns (3).

It has been postulated that the epithelium of origin is derived from one of the following sources:

1- Cell rests of enamel organ.

2- Epithelium of odontogenic cysts.

3- Disturbances of developing organ.

4- Basal cells of surface epithelium.

5- Heterotrophic epithelium in other parts of body (4).

It was first described by Broca in 1868 and constitutes 1% to 2% of all cysts and tumors of the jaws (5). It is prevalent in the 4th decade of life, but is seen in the age range from 6 months to 76 years (3). Clinically, it frequently manifests as a painless swelling, which can be accompanied by facial deformity, malocclusion, loss of dental pieces, ulceration and periodontal disease (6).

Ameloblastomas are divided into four categories based on radiological appearance, histological features, anatomic location: Unicystic, Multicystic or Solid, Desmoplastic, Peripheral (7). Histopathologic variants of ameloblastoma include follicular, plexiform, acanthomatous, granular cell, desmoplastic and basal cell patterns (8).

The Granular Cell Ameloblastoma (GCA) is one of the rarest entities and accounts for only 5% of all ameloblastomas (5).

The purpose of this article is to present a case of unusual variant of ameloblastoma and highlighting its unique microscopic features that allow its distinction from other jaw tumors with a granular cell consistency.

## Case Report

A 55 year old female reported with a painful swelling in the lower left back tooth region since 1 week. Patient was asymptomatic 1year back then noticed small swelling which was initially pea-nut in size and progressed to present size. There was no contributory past medical history.

**Figure 1:**
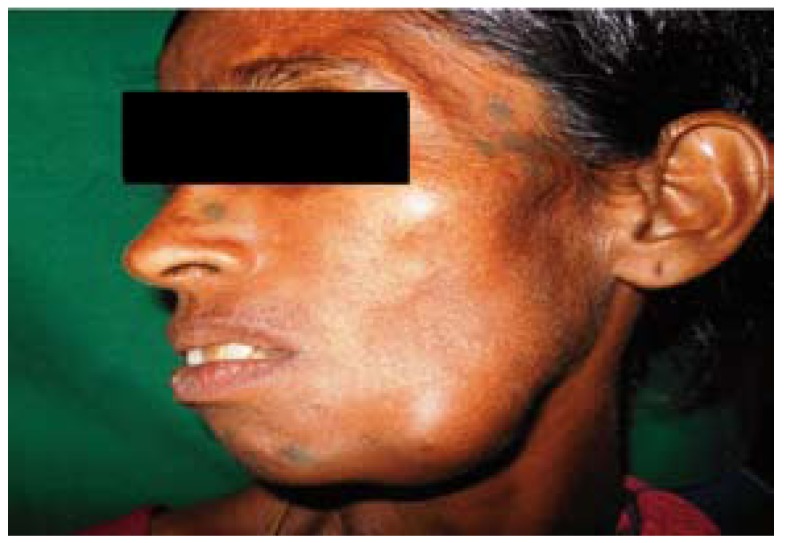
Extra oral picture showing swelling on left side of the mandible.

Extraorally, facial asymmetry was noted on left side of face. The swelling was 5x5cm in size approximately extending antero-posteriorly from parasymphysis to angle of mandible and supero-inferiorly 4cm from lower canthus of eye to inferior border of mandible on left side (Fig. 1). Tender on palpation and is firm in consistency. Two submandibular lymph nodes on either side are palpable which are approximately 0.8x0.6cm in size and are oval, fixed, tender and firm.

Intraoral examination revealed a diffuse swelling in the mandibular posterior region on left side extending along the buccal vestibule extending from 35 to 38 which was irregular in shape, pale pink in color, stony hard in consistency and associated with tenderness. Obliteration of the buccal vestibule was seen in relation to 36. Missing teeth in relation to 37, 38.

Aspiration of the lesion revealed reddish brown fluid. Orthopantamograph revealed an ill-defined radiolucent area extending from 36 to angle of mandible with a discontinuity in lower body of mandible on left side. A provisional diagnosis of intraosseous carcinoma of mandible was given.

**Figure 2:**
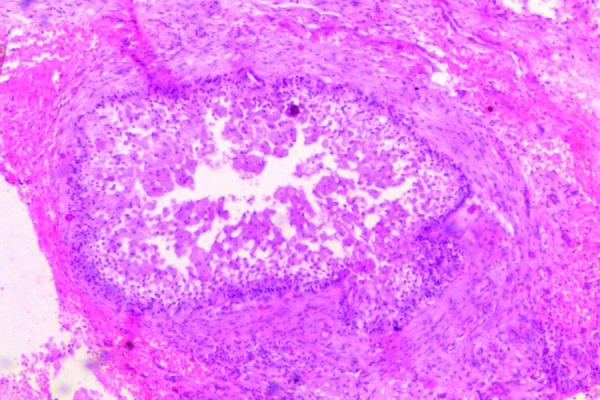
10x view showing ameloblastomatous follicle within fibrous connective tissue stroma.

**Figure 3:**
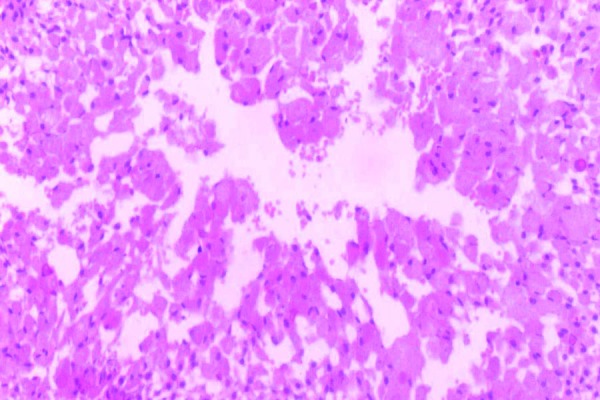
20x view showing the polygonal tumor cells having eosinophilic granular cytoplasm with eccentrically placed nuclei.

Incisional biopsy was sent for histopathological examination. The section shows ameloblastomatous follicle within fibrous connective tissue stroma (Fig. 2). Follicles shows peripheral tall columnar cells and central stellate reticulum like cells showing granularity in the cytoplasm. Nuclear atypia is seen in few areas of connective tissue stroma (Fig. 3). Focal areas of necrosis and hemorrhage are evident. The final diagnosis of Granular Cell Ameloblastoma was given.

## Discussion

Ameloblastomas sometimes exhibit granular transformation of cytoplasm, usually occurring in central stellate reticulumlike cells, and this change often extends to peripheral columnar or cuboidal cells (9).

Numerous theories have been proposed on the origin and nature of these granular cells in ameloblastomas. These granular cells are epithelial in origin and several ultrastructural and histochemical studies have described them as lysosomes (5). Lysosomal aggregation within the cytoplasm is caused by dysfunction of either a lysosomal enzyme or lysosome-associated protein involved in enzyme activation, enzyme targeting, or lysosomal biogenesis (10).

Granular cells are just a transitional or matured phase in the lifecycle of ameloblastomas, starting with normal stellate reticulum like cells leading to production of granules and finally leading to degeneration and formation of cystic areas (5).

It is evident from the literature, there exist two main lines of interpretation in that some consider it as a metabolic, whilst others of the view that it represent a degenerative process. More recent observation support the later view to be more tenable based on the increased expression of death signaling molecules (3). Ara et.al., suggested that the synthesis of signaling molecules, such as β-catenin and Wnt-5a is upregulated in the granular cells of GCA, but their transportation or secretion is impaired, resulting their accumulation within granular cells, as autophagosomes (10).

Histopathologically GCA has numerous large eosinophilic granular cells. These cells usually form the central mass of the epithelial tumor islands and cords. The periphery of the islands consists of non-granular tall columnar cells (3).

Immunohistochemically granular cells are positive for CD68, Lysozyme and α1 antichymotrypsin but negative for vimentin, desmin, S-100, neuron specific enolase and CD15, indicating cytoplasmic lysosomal aggregates not of mesenchymal, myogenic or neurogenic origin. A recent immunohistochemical and ultrastructural study by Kumamoto et al., suggests that the cytoplasmic granularity might be attributed to the increased apoptotic cell death of the neoplastic granular cells and their subsequent phagocytosis by the adjacent granular cells (9). The strong expression of basement membrane proteins, including laminins 1 and 5 and fibronectin, was observed in many granular cells of ameloblastoma, providing some insights into the characteristics of granular cells in this rare tumor (8).

Differential diagnosis of GCA includes other oral lesions with a similar morphology of granular cell accumulation including granular cell tumor and congenital epulis.

Complete surgical excision is the treatment of choice. Ameloblastoma is a tumor that frequently recurs after treatment. The rate of recurrence ranges from 4.5% for enbloc resection to 54.1% for conservative therapy (3).

## Conclusion

GCA is a rare condition with unique histopathologic and immunohistochemical findings. GCA should be differentiated from the other variants of ameloblastoma and also from other granular cell lesions because of its high recurrence rate. Patients should be kept under periodic observation because of reports of recurrences even up to 8yrs after initial treatment.
